# Correction: Surgical and regional treatments for colorectal cancer metastases in older patients: A systematic review and meta-analysis

**DOI:** 10.1371/journal.pone.0251005

**Published:** 2021-04-27

**Authors:** Nicola de’Angelis, Capucine Baldini, Raffaele Brustia, Patrick Pessaux, Daniele Sommacale, Alexis Laurent, Bertrand Le Roy, Vania Tacher, Hicham Kobeiter, Alain Luciani, Elena Paillaud, Thomas Aparicio, Florence Canoui-Poitrine, Evelyne Liuu

The thirteenth author’s name is spelled incorrectly. The correct name is: Florence Canoui-Poitrine.
