# Supplementary figures and images for: Surgical and regional treatments for colorectal cancer metastases in older patients: A systematic review and meta-analysis
Source: PLoS One. 2020 Apr 22;15(4):e0230914. doi: 10.1371/journal.pone.0230914 (PMC7176093; doi:10.1371/journal.pone.0230914)

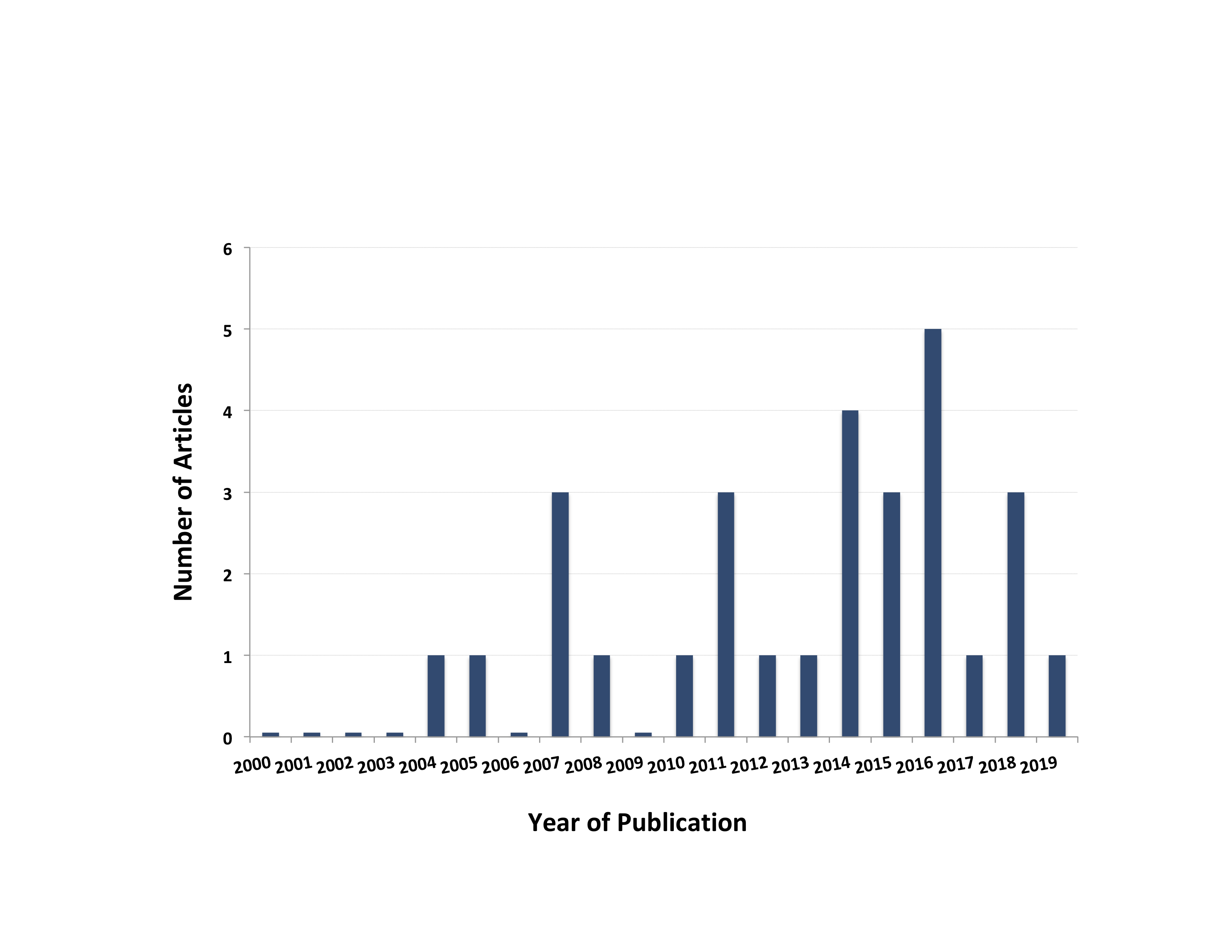

Supplement: S1 Fig — (TIFF) [file pone.0230914.s002.tiff]

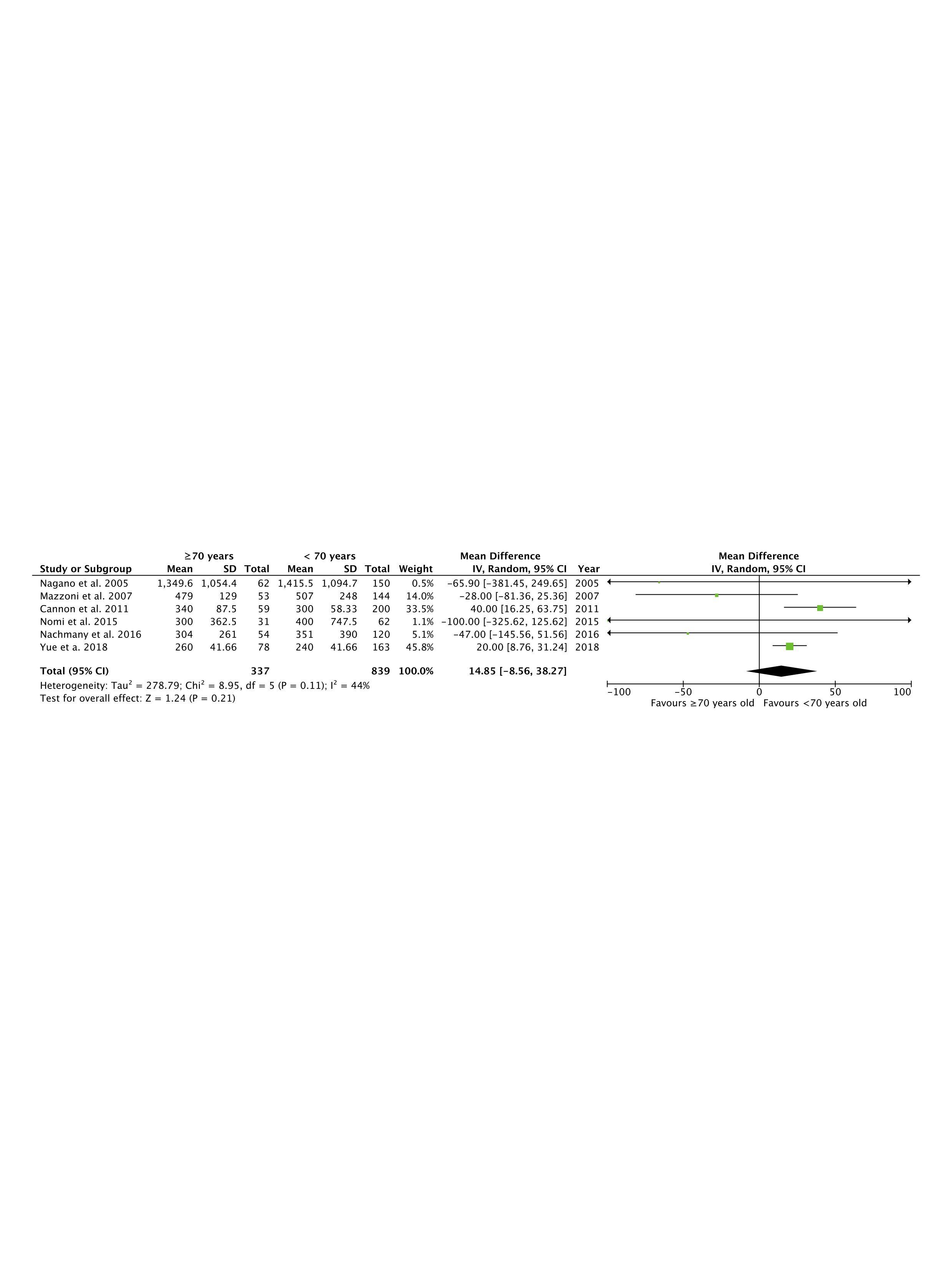

Supplement: S2 Fig — (TIFF) [file pone.0230914.s003.tiff]

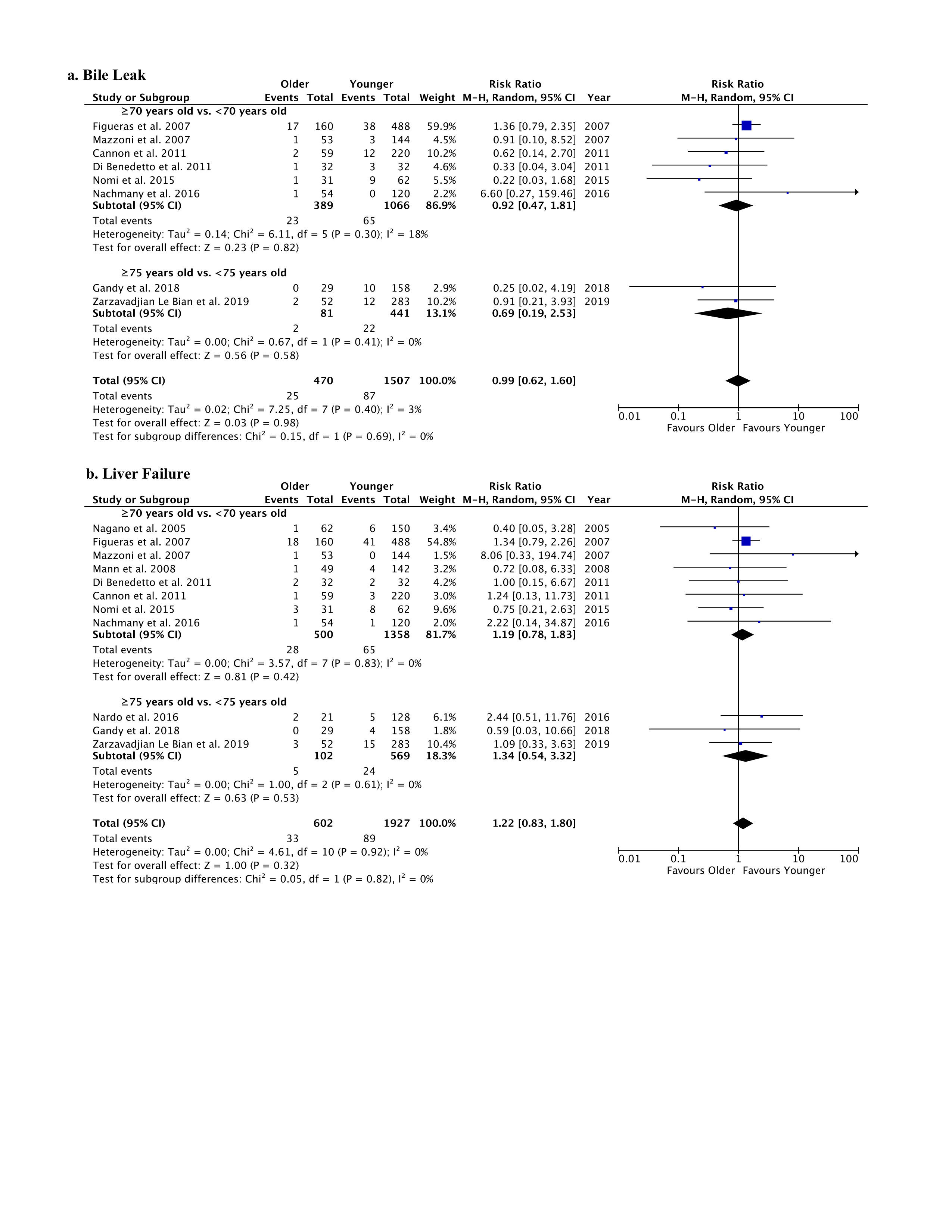

Supplement: S3 Fig — The following outcomes were analyzed: a. bile leak (n) and b. liver failure (n). (TIFF) [file pone.0230914.s004.tiff]

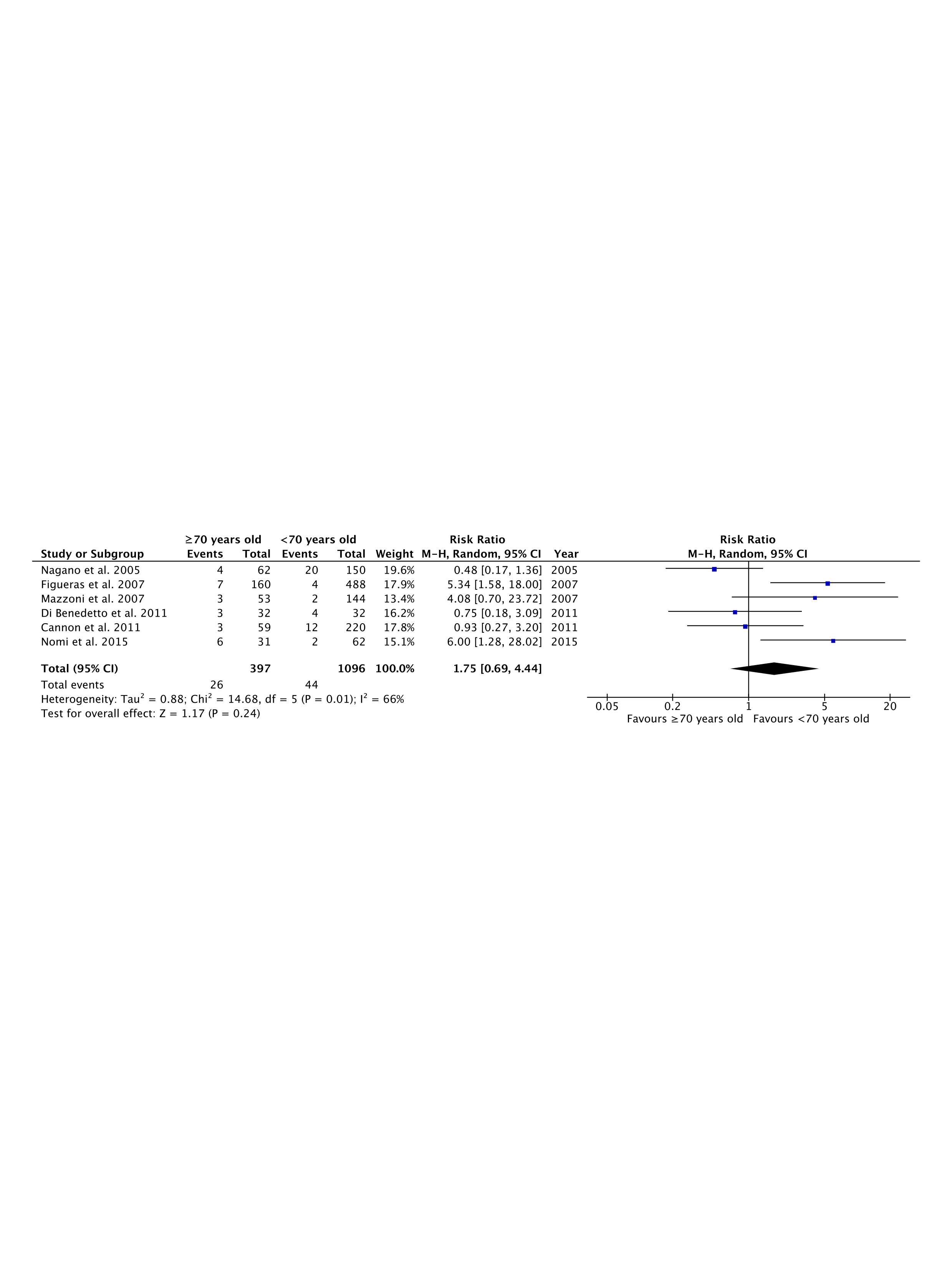

Supplement: S4 Fig — (TIFF) [file pone.0230914.s005.tiff]

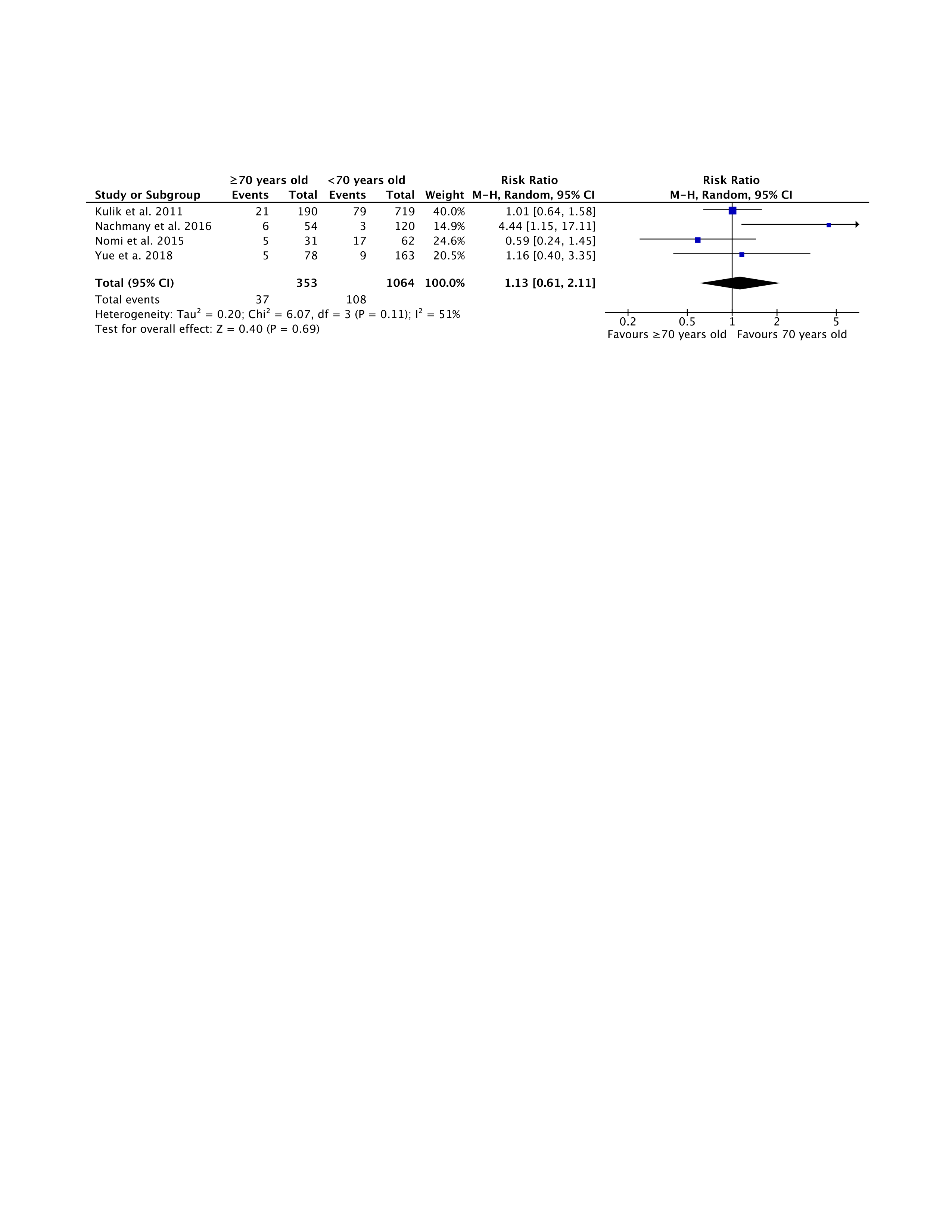

Supplement: S5 Fig — (TIFF) [file pone.0230914.s006.tiff]

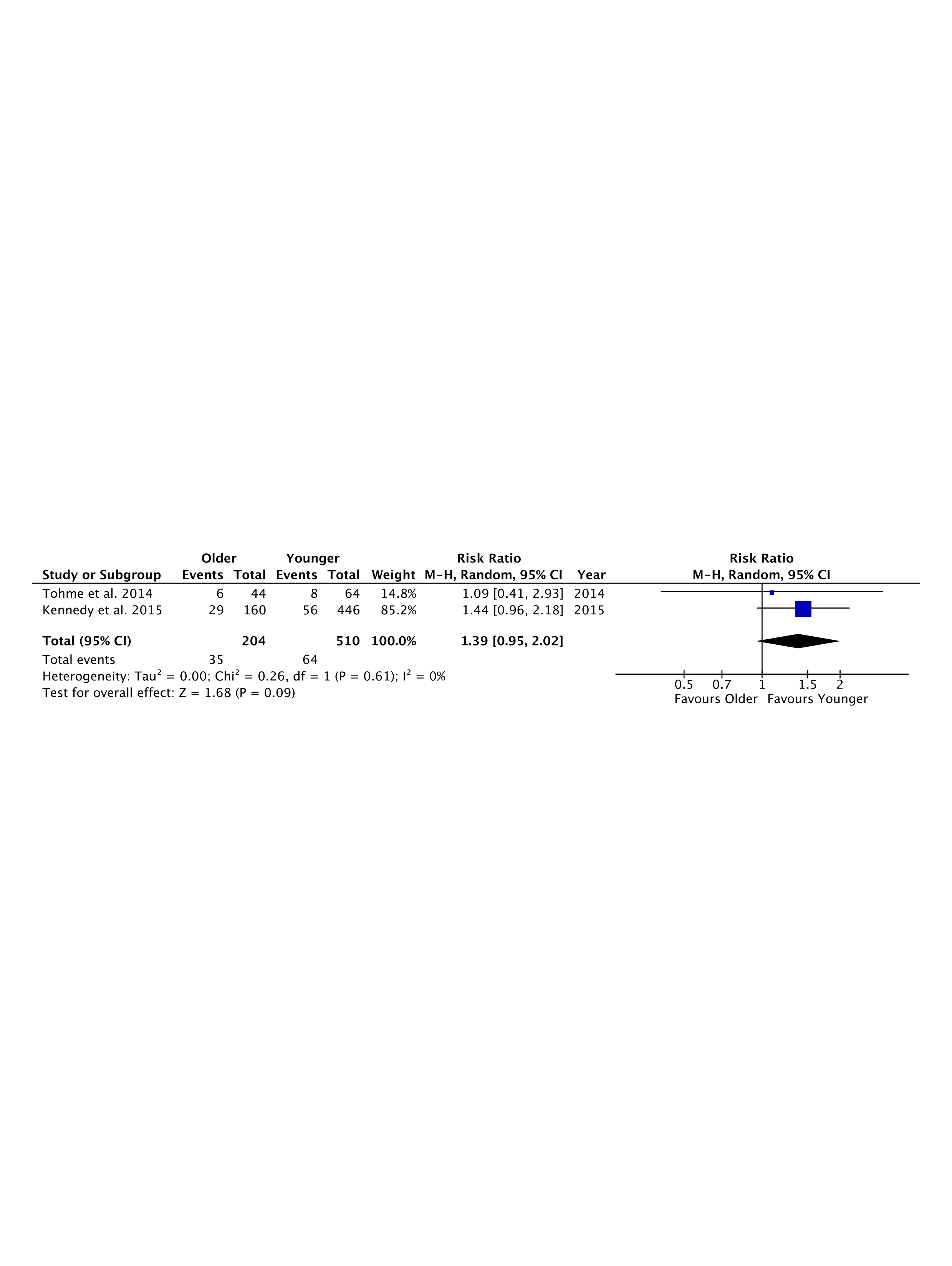

Supplement: S6 Fig — (TIFF) [file pone.0230914.s007.tiff]
