# Supplementary material for: Surgical and regional treatments for colorectal cancer metastases in older patients: A systematic review and meta-analysis
Source: PLoS One. 2020 Apr 22;15(4):e0230914. doi: 10.1371/journal.pone.0230914 (PMC7176093; doi:10.1371/journal.pone.0230914)
Supplement: S1 Table — (DOCX) [file pone.0230914.s008.docx]

**Supplemental Table 1. Study quality assessment using Newcastle-Ottawa scale (NOS).**

| **Ref** | **Selection** | **Comparability** | **Outcome / Exposure** | **Overall Score** |
| --- | --- | --- | --- | --- |
| **Zacharias et al. 2004** | ******* | **-** | ***** | **4** |
| **Nagano et al. 2005** | ******* | **-** | ***** | **4** |
| **Cummings et al. 2007** | ******** | ***** | ****** | **7** |
| **Figueras et al. 2007** | ******* | ***** | ***** | **5** |
| **Mazzoni et al. 2007** | ******* | ***** | ****** | **6** |
| **Mann et al. 2008** | ******* | **-** | ***** | **4** |
| **Adam et al. 2010** | ******** | ***** | ***** | **6** |
| **Di Benedetto et al. 2011** | ******* | ***** | ****** | **6** |
| **Cannon et al. 2011** | ******* | ***** | ****** | **6** |
| **Cook et al. 2011** | ******** | ***** | ****** | **7** |
| **Kulik et al. 2011** | ******* | **-** | ****** | **5** |
| **Kumar et al. 2013** | ******** | **-** | ***** | **5** |
| **Doat et al. 2014** | ******** | ***** | ****** | **7** |
| **Khan et al. 2014** | ******* | ***** | ****** | **6** |
| **Nomi et al. 2014** | ******* | **-** | ****** | **5** |
| **Tohme et al. 2014** | ******* | ***** | ****** | **6** |
| **Booth et al. 2015** | ******** | ****** | ****** | **8** |
| **Kennedy et al. 2015** | ******* | **-** | ****** | **5** |
| **Parakh et al. 2015** | ******* | ***** | ****** | **6** |
| **Grande et al. 2016** | ****** | **-** | ****** | **4** |
| **Massarweh et al. 2016** | ******** | ****** | ****** | **8** |
| **Nachmany et al. 2016** | ******* | **-** | ***** | **4** |
| **Nardo et al. 2016** | ******* | ***** | ***** | **5** |
| **Zheng et al. 2016** | ****** | ****** | ****** | **6** |
| **Martinez Cecilia et al. 2017** | ******** | ***** | ****** | **7** |
| **Gandy et al. 2018** | ******* | **-** | ***** | **4** |
| **Yue et al. 2018** | ******* | ***** | ****** | **5** |
| **Zarzavadjian Le Bian et al. 2019** | ******** | **-** | ***** | **5** |
